# Endoreplication and its consequences in the suspensor of *Pisum sativum*

**DOI:** 10.1007/s00299-018-2335-0

**Published:** 2018-08-21

**Authors:** Agnieszka Chmielnicka, Aneta Żabka, Konrad Krajewski, Janusz Maszewski, Justyna Teresa Polit

**Affiliations:** Department of Cytophysiology, Faculty of Biology and Environmental Protection, University of Łódź, Pomorska 141/143, 90-236 Łódź, Poland

**Keywords:** Embryo, Endoreplication, Suspensor, Transcription

## Abstract

**Key message:**

DNA replication and continuous process of transcription during ongoing amitotic division accelerate the development of four-celled pea suspensor containing nuclei which create transient gradient of polyploidy necessary for correct embryo development.

**Abstract:**

A suspensor, the link between embryo proper and surrounding tissues, differs significantly in size, morphology, and degree of polyploidy among the species. The suspensor of *Pisum sativum* consists of four polynuclear cells (two hemispherical and two elongated) formed in two layers. Their nuclei undergo endoreplication reaching, respectively, up to 256C and 128–256C DNA levels in its hemispherical and elongated parts. Our study shows that endoreplication first appears in the spherical part of the suspensor, and, subsequently, in the elongated one. At the next stages of suspensor development, the increase in DNA content takes place also in a similar order. Thus, despite simple construction of the suspensor, its development, supported by endoreplication, creates a certain gradient of polyploidy, which occurs in more extensive suspensors. Moreover, the rapid development of suspensor is supported both by the initiation of DNA replication prior to the completion of amitotic division of its polyploidal nuclei and by a continuous process of transcription, which is silenced by chromatin condensation throughout mitosis. Furthermore, the increase in DNA content correlates with the greater amount of transcripts; however, the multiplication of DNA copies does not entail an increase (but fluctuation) in the mean transcriptional activity of a particular nucleus during the next stages of suspensor development.

## Introduction

In almost all plants, the first division of a typical zygote (polarized along its micropylar–chalazal axis) takes place in a perpendicular plane to the long axis of the cell, and most often, it is asymmetric, resulting in a high basal cell and a smaller apical cell. An embryo proper develops mainly from the apical cell, while the suspensor or its part is predominantly formed from the basal cell. However, considering the plan of the first division of an apical cell and the role of the basal cell in embryo ontogenesis, six different types of embryogenesis were distinguished in plants: *Onagrad, Asterad, Caryophyllad, Chenopodiad, Solanad*, and *Piperad* (Raghavan 1997; Souter and Lindsey 2000; Long 2006). The mechanism which directs the basal cell to develop into the suspensor may be based on the transcription of different genes, but it is still an unsolved problem. Weterings et al. (2001) demonstrated, for example, that transcription products of C541 and G564 genes of *Phaseolus coccineus* were present in two derivatives of the basal cell, giving rise to the suspensor, while they did not occur in the apical cells. Regulation of gene expression in the suspensor is now the subject of many studies, which may help to better understand the molecular mechanisms of differentiation of this structure (Weterings et al. 2001; Kawashima et al. 2009; Henry and Goldberg 2015; Henry et al. 2015; Peng and Sun 2018; Xie et al. 2018).

The suspensor occurs in most angiosperms species. Structures with similar features and functions are also present in gymnosperms and lower plants. Suspensors significantly differ in size and morphology among the species. They can be unicellular or multicellular, small or large in relation to the embryo proper, columnar, spherical, or irregularly shaped, and their cells may be multinuclear or may produce haustoria. In some species, the border between the suspensor and the embryo proper is difficult to determine (Kawashima and Goldberg 2010).

**Fig. 1:**
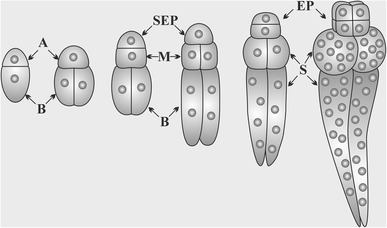
Scheme of the first few divisions of the zygote of *Pisum sativum*;* A* apical cell of zygote and* B* bazal cell of zygote and then its derivatives, *M* middle cell, *SEP* the stem cell of embryo proper, *EP* embryo proper, *S* suspensor

The suspensor in *Pisum sativum* (*Solanad* type embryogenesis) consists of four multinucleate cells arranged in two layers, and develops from the middle and basal cells (Fig. 1), which originated from the asymmetric division of the polarized zygote (Raghavan 1997; Souter and Lindsey 2000; Long 2006). The middle cell, adjacent to the embryo, divides into two hemispheres, in which the embryo proper is embedded. Longitudinal division of the basal cell produces two considerably elongated suspensor cells, extending to the micropylar region of the embryo sac (Cooper 1938; Liu et al. 1996).

**Fig. 2:**
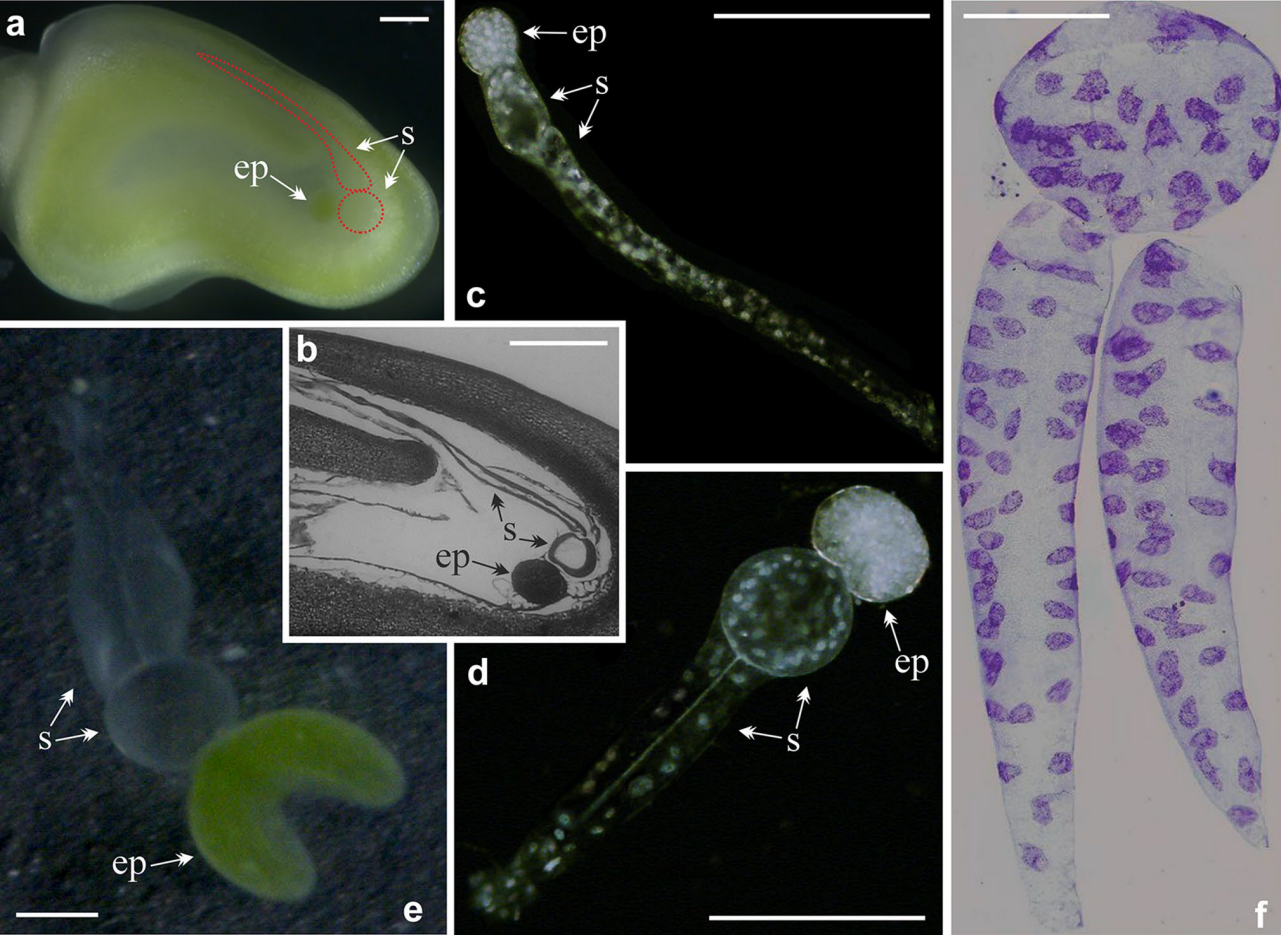
Suspensor of *P. sativum* var. Paloma. **a** Longitudinal section of a seed under the light microscopy. Faintly visible suspensor—s, schematically marked dotted outline in the vicinity of the globular embryo proper—ep. **b** Semithin longitudinal section of a seed stained with haematoxylin. Clearly visible arrangements of the suspensor—s and the globular embryo proper—ep. **c**–**e** Isolated suspensors—s with embryos proper—ep in the early globular stage **c**, mid-globular stage **d**, very early cotyledon stage **e** observed in dark-field microscopy **c, d** and light microscopy **e. f** Suspensor with Feulgen-stained endopolyploid nuclei. Scale bar 250 µm

In angiosperms, the suspensor is a part of the embryo which quickly differentiates and significantly overtakes the development of the embryo proper (Fig. 2a, b). It becomes mature when the embryo proper is still in the pro-embryonic state (Marinos 1970a, b; Lersten 1983). Although the suspensor is a short-lived structure, it fulfils an important role in embryogenesis forming a kind of link between the embryo proper and the surrounding tissues. It supplies nutrients and hormones to the embryo, as well as maintains the embryo proper in the right position, pushing it into the endosperm tissue and directing the embryonic root tip (Marinos 1970a, b; Umehara and Kamada 2005; Kawashima and Goldberg 2010). The function of the suspensor is reflected in its large four-celled multinuclear structure (syncytium), which greatly facilitates rapid transport of substances to the embryo (cell walls are reduced to minimum). Transport of substances to the embryo occurs via symplast. Between multinuclear cells of the suspensor, as well as between the suspensor and the embryo proper, there are numerous plasmodesmata, which very rarely connect the suspensor with the other parts of a seed (Marinos 1970a, b; Johansson and Walles 1994). Most likely, nutrients from the endosperm are delivered to the suspensor along the apoplastic pathway (Stadler et al. 2005).

During the early embryogenesis, metabolic activity of the suspensor is high, much higher than the metabolic activity of the embryo proper. Probably, during this period, the suspensor supplies the embryo proper with components essential for its development (Lersten 1983; Kawashima and Goldberg 2010; Peng and Sun 2018).

Suspensors have invaginated cell walls, characteristic of transfer cells. They facilitate the transport of dissolved substances by increasing the contact surface of cells. This kind of cell walls was described in suspensors of numerous plant species, including *Phaseolus, Capsella, Arabidopsis, Epilobium, Antirrhinum, Lathyrus, Scrophularia, Stellaria, Diplotaxis, Tropaeolum, Alyssum, Medicago, Alisma, Paphiopedilum, Sedum, Sempervivum, Jovibarba, Eruca*, and *Chenopodium* (Alessio et al. 2010; Kozieradzka-Kiszkurno et al. 2012; López-Fernández and Maldonado 2013). In *Pisum sativum* and in *Vicia faba*, the suspensors have a very similar construction with cell wall ingrowths along their surfaces, which allow them to maintain contact with endosperm (Marinos 1970a, b; Johansson and Walles 1994). However, such protuberances do not always occur over the whole surface of the suspensor and are often limited to its basal part, most distant from the embryo proper. In many representatives of *Crassulaceae, Fumariaceae, Rubiaceae, Trapaceae*, or *Tropaeolaceae* families, the suspensor forms structures called haustoria. They penetrate adjacent cells; for example, endosperm, nucellus, or integuments, and draw nutrients for the developing embryo proper from them. In some species (e.g., *Crassulaceae*), a huge basal cell plays the role of haustorium (Kozieradzka-Kiszkurno et al. 2011, 2012; Czaplejewicz and Kozieradzka-Kiszkurno 2013).

The energy required for active transport of substances is provided by numerous mitochondria located close to the invaginations of a suspensor cell wall (Marinos 1970a, b; Yeung and Meinke 1993; Kawashima and Goldberg 2010; Kozieradzka-Kiszkurno et al. 2011). Suspensor cells also contain a large amount of specialized plastids and endoplasmic reticulum. The polyploidy of suspensor nuclei and the possible presence of polytene chromosomes may be connected with the ability to synthesize large quantities of various substances (including plant hormones) necessary for the growth and development of the embryo proper (Marinos 1970a, b; Yeung and Meinke 1993; Larsson et al. 2008; Kawashima and Goldberg 2010). Suspensors of many plant species produce gibberellins (Wredle et al. 2001; Kawashima and Goldberg 2010). Furthermore, the presence of auxins, cytokinins, and abscisic acid was also reported, but it is not certain whether all these substances are synthesized inside these structures. Hormones are important not only for the embryo proper development, but also for the suspensor. It is believed that auxins help to maintain the identity of the suspensor cells. In *Arabidopsis thaliana*, in the hypophysis (the suspensor cell nearest the embryo), auxin causes cellular specification and mediates asymmetric division, while, in the subsequent cells, it prevents their proliferation and transformation into the embryo proper (Umehara and Kamada 2005; Rademacher et al. 2012). During the development of cotyledons, the suspensor begins to degenerate and is usually absent from mature seeds (Lersten 1983; Lombardi et al. 2007). The suspensor disappears as a result of genetically determined programmed cell death (PCD; Umehara and Kamada 2005), which helps organisms to remove structures that already fulfilled their functions (Elmore 2007; Lombardi et al. 2007).

Despite general similarities in the development of embryos and whole seeds, different species of the same family (e.g., *Fabaceae*), as well as cultivars occurring within a single species (e.g., *Pisum*) show significant differences in the degree of endopolyploidization and distribution of endopolyploidal cells within the embryo.

Thus, the objectives of this study were to evaluate the dynamics and the degree of nuclear DNA endoreplication at the successive stages of suspensor development and to determine whether the intensity of transcription increases together with nuclear ploidy.

The presented study indicates that inside characteristic four-celled structure of *P. sativum* suspensor, endoreplication begins very early, gradually increases during the development (forming periodic gradient of nuclear DNA content in two layers of the suspensor), and, in its final stage, creates nuclei with high ploidy level (128-256C) distributed both in the spherical and in the elongated parts of the suspensor. The multiplication of DNA copies is correlated with the greater amount of transcripts; however, it does not lead to linear increase in the average intensity of transcription in the area of particular nuclei at the subsequent stages of development, whereas acceleration of the suspensor growth and development is significantly affected by phenomena showing some coincidence in large polyploid nuclei (endoreplication, transcription, and amitotic divisions).

## Materials and methods

### Plant material

Suspensors were isolated from the seeds developed in pea pods (*Pisum sativum* var. Paloma), derived from a potted cultivation carried out at 26 °C, and the 170 µE/m^2^/s light intensity and 16/8 h (day/night) photoperiod. The terms used to describe developmental stages of the suspensor are based on those used to denote successive stages of the embryo proper.

### Feulgen staining and cytophotometry

Pea pods at successive stages of development were fixed for 24 h in the mixture of methanol, formalin, and glacial acetic acid (MAF; 80:15:5 v/v; room temperature). After fixation, isolated seeds were rinsed in 70% methanol, rehydrated (70–10% ethanol, distilled water, 5 min each), hydrolyzed in 4M HCl (1 h), stained with Schiff’s reagent (pararosaniline; Sigma-Aldrich; 1 h), and washed with SO_2_ water (three times) and distilled water. Using a stereoscopic microscope PZO HSt 130 with an ocular scale, the suspensors at successive stages of development were isolated and squashed in a drop of water onto slides (Polysine™ Menzel Glaser) using the dry ice method. After removing the cover slips, slides were plunged into 70% ethanol, air dried, and mounted in Canada balsam. Nuclear DNA content was evaluated by means of microdensitometry using Optifot-2 microscope (Nikon, Japan) with the computer-aided cytophotometer (IMAL1024, Poland). The extinction of Feulgen-stained cell nuclei was measured at 550 nm and calibrated in arbitrary units, taking the values recorded for half-telophases and prophases from the embryo proper as the reference standard of 2C and 4C DNA levels, respectively.

### Labeling and detection of RNA transcripts

Click-iT^®^ RNA Alexa Fluor^®^ 488 Imaging Kit (Invitrogen) was used for the visualization of RNA transcripts (Jao and Salic 2008). Isolated seeds were placed in 2 mM water solution of 5-ethynyluridine (5-EU) in the dark. After 2 h labeling, the seeds were rinsed in distilled water and fixed in PBS-buffered 4% paraformaldehyde (4 °C; pH 7.2) for 45 min. After rinsing in PBS, the suspensors were isolated (as for cytophotometry), squashed onto microscope slides (Polysine™,Menzel-Gläser) in a drop of distilled water, and placed on dry ice. After 5 min, cover slips were removed; the slides were washed with PBS, distilled water, and air dried. Then, the suspensors were permeabilized with 0.5% Triton X-100 for 15 min and rinsed with PBS. Sites of EU incorporation were detected using Click-iT^®^ reaction cocktail consisting of components prepared according to the vendor’s manual. Incubation was performed in the dark for 60 min at room temperature. Then, the slides were washed with Click-iT® reaction rinse buffer and PBS. Cell nuclei were additionally stained with 1 µM DAPI for 5 min and washed in PBS. The samples mounted in PBS/glycerol mixture (9:1) containing 2.5% DABCO were photographed at exactly the same time of integration using the Eclipse E600W microscope (Nikon) and DS-Fi1 CCD camera (Nikon). For Alexa Fluor^®^ 488 and for DAPI the DM 505 filter (excitation wavelength, 465–495 nm) and the DM 400 filter (excitation wavelength, 340–380 nm) were used, respectively. Quantitative measurements of fluorescence were made after converting color images into gray scale and expressed in arbitrary units as mean pixel values (pv) spanning the range from 0 (dark) to 255 (white). At each stage of the suspensor development, 1000 cell nuclei were analyzed. The obtained data were expressed as the mean values ± standard deviation of the mean (SD), and Tukey’s *t* tests were used to compare individual variables.

### Labeling and detection of DNA replication

Click-iT^®^ DNA Alexa Fluor^®^ 555 Imaging Kit (Invitrogen) was used for the visualization of DNA replication. Isolated seeds were placed in 20 µM water solution of 5-ethynyl-2′-deoxyuridine (EdU) in the dark. After 0.5 h labeling, the seeds were rinsed with distilled water and fixed in PBS-buffered 4% paraformaldehyde (pH 7.4; 4 °C) for 45 min. After rinsing with PBS containing 3% BSA, the suspensors were isolated (as for cytophotometry) squashed onto microscope slides (Polysine™, Menzel-Gläser) in a drop of buffer, and placed on dry ice. After 5 min, cover slips were removed, and the slides were washed with PBS, distilled water, and air dried. Then, the suspensors were permeabilized with 0.5% Triton X-100 for 15 min and rinsed with PBS containing 3% BSA. Sites of EdU incorporation were detected using Click-iT^®^ reaction cocktail consisting of components prepared according to the vendor’s manual. The procedure was performed in the dark for 30 min at room temperature. Next, the slides were washed with PBS containing 3% BSA and then in PBS. Cell nuclei were counterstained with 1 µM DAPI for 5 min and washed in PBS. The samples mounted in PBS/glycerol mixture (9:1) containing 2.5% DABCO were photographed using the Eclipse E600W microscope (Nikon) and DS-Fi1 CCD camera (Nikon). For Alexa Fluor^®^ 555 and for DAPI, the DM 565 filter (excitation wavelength, 540/25) and the DM 400 filter (excitation wavelength, 340–380 nm) were used, respectively.

### Hematoxylin staining

For the photographic documentation, the material was embedded in paraffin by the standard method, and sectioned and stained with Heidenhain’s iron haematoxylin according to Bio-Optica protocol (Milano-Italy).

## Results

**Fig. 3:**
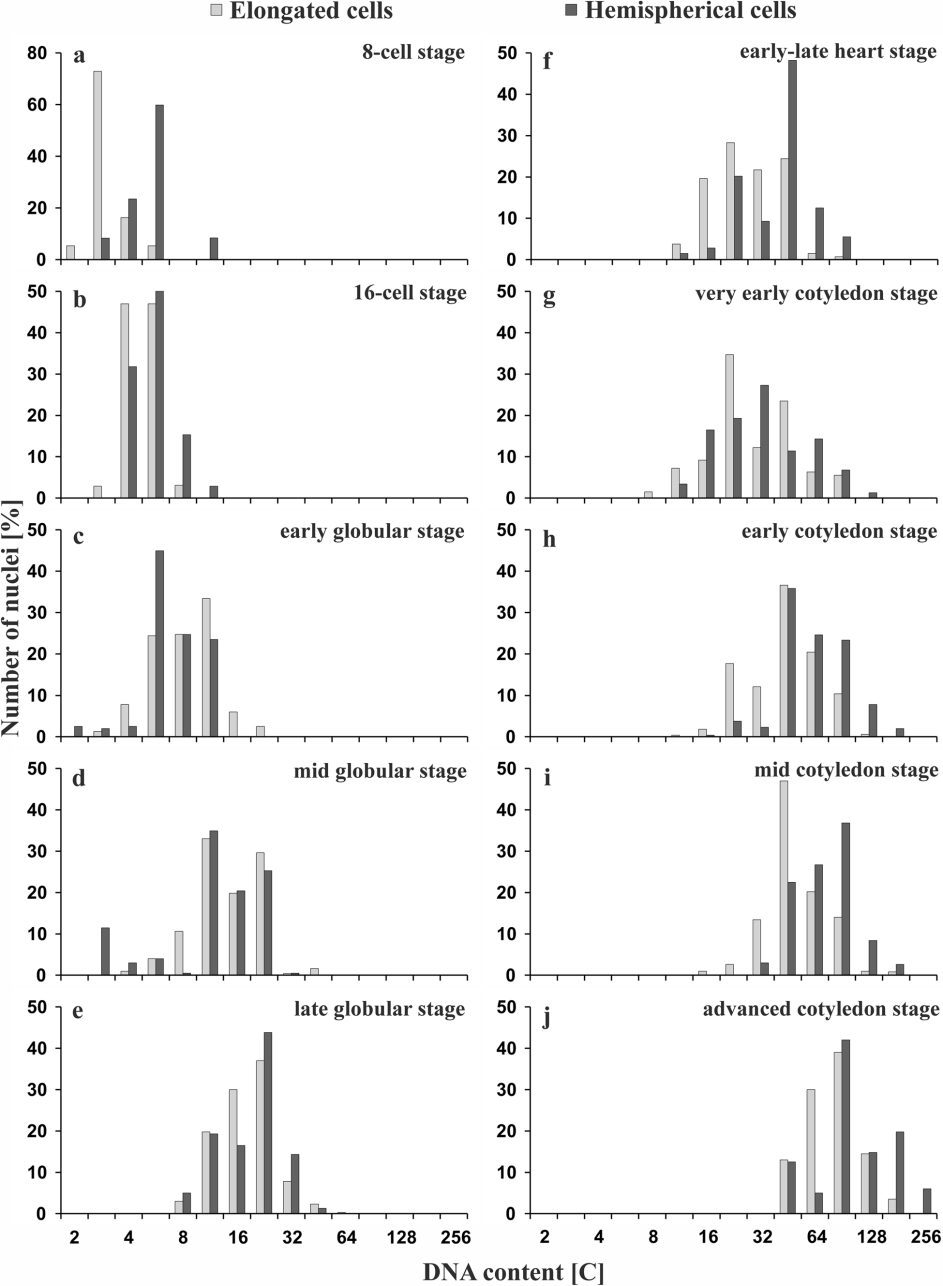
Changes in the nuclear DNA content of the elongated and spherical part of suspensor during the successive stages of *P. sativum* var. Paloma seed development. **a–j** Developmental stages of the embryo proper (accompanied by the suspensor) are shown in the fields of individual diagrams. In each experimental series, five suspensors were analyzed and the studies were repeated three times

During the development of *Pisum sativum* var. *Paloma* seeds (Fig. 2a, b), four morphologically distinct stages of the embryo proper can be discerned. Accordingly, successive stages of suspensor development were assigned as follows: (1) the proembryo stage, characterized by a few cells; (2) the globular stage during which the embryo proper takes a spherical shape (Fig. 2a–d); (3) the heart-shaped stage, during which one of the poles forms characteristic invagination; (4) the cotyledon stage, during which a definite axis of the embryo connects two separate cotyledons (Fig. 2e); the classical torpedo stage does not occur. The duration of particular phases varies: some are extremely short (e.g., proembryo stage); others take a long time, within which dynamic functional changes take place (e.g., at the cotyledon stage). Consequently, more precise classification of stages was made. The proembryo stage was divided into 8-cell and 16-celled sub-stages, the globular stage into early, mid, and late ones, the heart stage into early and late sub-stages, and the cotyledon stage into very early, early, mid, and advanced ones. In the latter sub-stage, the embryo becomes approx. 2.5 mm in diameter. During later stages of embryo development, the suspensor undergoes degradation.

### Changes of the DNA content in the suspensor nuclei

The suspensor cells of *Pisum sativum* are multinucleate. Each of the two hemispherical cells contains 32 nuclei, and each of the two elongated cells contains 64 nuclei, showing different levels of endopolyploidy. To determine the changes of DNA content in the nuclei from both structural parts of the suspensor (spherical and elongated), cytometric analyses of nuclei stained with Feulgen reaction were performed (Fig. 2f). In some cases (if there was no difference between the DNA contents in the nuclei at two different stages), the data are presented jointly (e.g., Fig. 3f).

DNA endoreplication in the suspensor begins very early, and first, it takes place in nuclei from its spherical part. At the eight-cell stage of the embryo proper, the nuclei containing 2C DNA were not observed in the spherical part of the suspensor (Fig. 3a). Only about 30% of the nuclei had the basic 2–4 and 4C DNA content, while the vast majority of the nuclei (approx. 60% passing through the first round of DNA replication out of the cell cycle) contained 4–8C DNA. A small pool of nuclei was also in the second round of endoreplication (8–16C DNA). During the 16-cell developmental stage of the embryo proper, in the spherical part of the suspensor, the distribution of nuclei was quite similar (Fig. 3b).

However, in the elongated part of the suspensor (at the 8-cell stage of the embryo proper), the vast majority of nuclei were still during interphase of the classic cell cycle, and only 5% of nuclei entered endoreplication (Fig. 3a). The number of nuclei during the first round of endoreplication increased to approx. 47% only after the embryo proper had reached the 16-cell stage; at that time, nuclei containing 8C DNA appeared for the first time (Fig. 3b).

At the early and mid-globular stage of the embryo proper, in both suspensor parts, 2, 2–4 and 4C DNA nuclei were rare (approx. 10%; Fig. 3c, d). They definitely disappeared when the embryo proper was at the late globular stage (Fig. 3e). During the subsequent steps of the globular stage, in both parts of suspensor, a similar fraction of cells (from 30 to 40%) passed the second and third round of endoreplication (Fig. 3c–e).

At the heart stage of the embryo proper, the nuclei with higher DNA contents were again more abundant in the spherical part of the suspensor than in the elongated one (Fig. 3f). In the former, nearly 50% of the cells were in the fourth round of endoreplication, over 10% completed it, and about 5% started the fifth round of the endocycle; whereas, in the elongated part of the suspensor, the fourth round of endoreplication just started and much fewer cells entered it (approx. 24%).

**Fig. 4:**
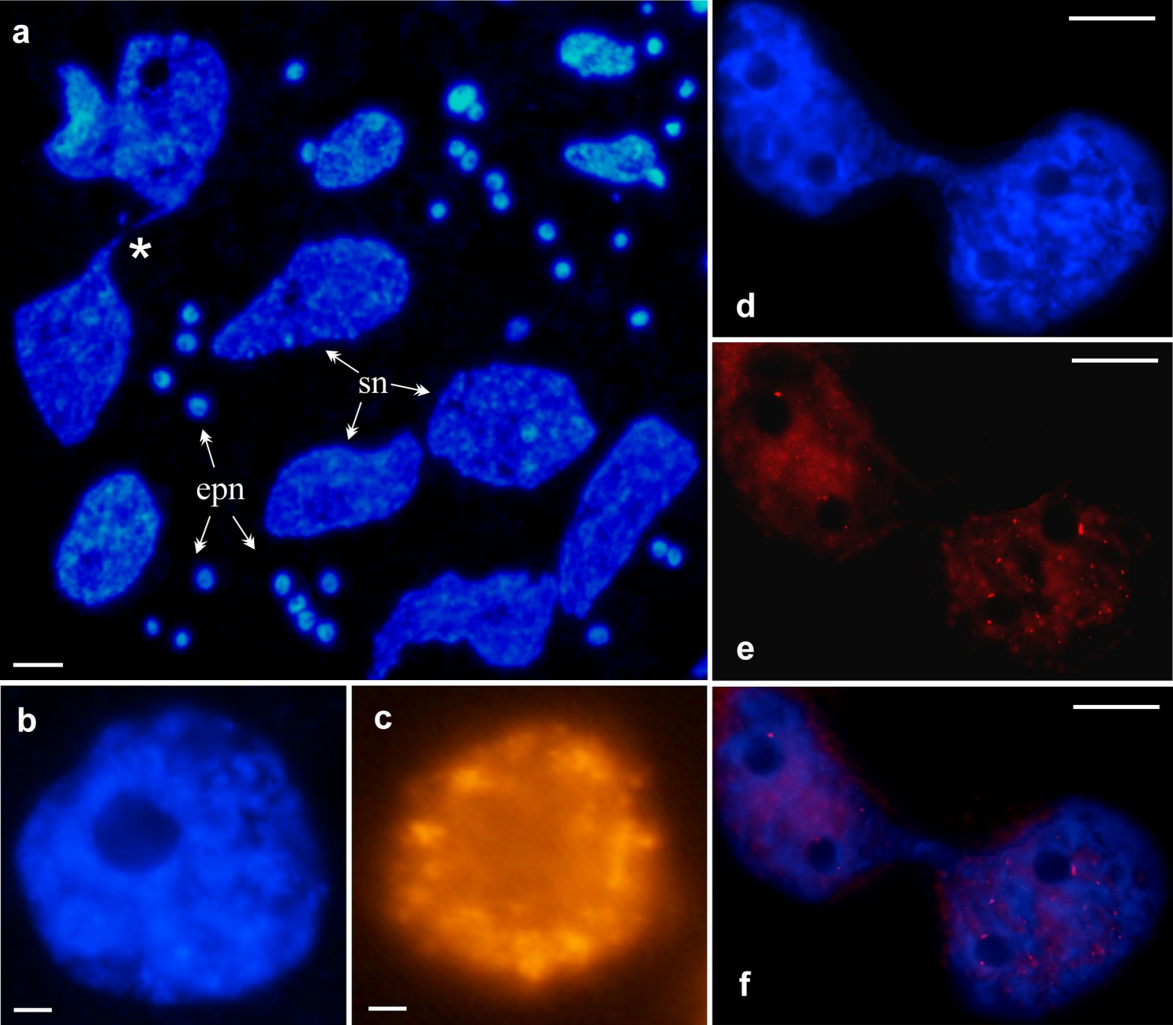
Endoreplication in the nuclei of *P. sativum* var. Paloma suspensor. **a** Endopolyploid nuclei of the suspensor—sn (dividing nuclei marked with an asterisk*) and the embryo proper nuclei—epn; DAPI staining. **b, c** Endopolyploid nucleus during replication stained with DAPI and 5′-ethynyl-2′-deoxyuridine (EdU) incorporation, respectively. **d**–**f** Endopolyploidal nucleus of the suspensor replicating DNA during division, stained with DAPI (**d**), EdU (**e**), and combined images **d** and **e** (**f**). Scale bar **a** − 50 µm; **b, c** − 5 µm; **d**–**f** − 25 µm

At a very early cotyledon stage, DNA content values were again similar in both structural parts of the suspensor (Fig. 3g). This resulted from the fact that, in the spherical part of the suspensor, the nuclei with a high degree of ploidy were dividing amitotically (see Fig. 4a), while, in the elongated part, endocycles continued.

At the early cotyledon stage, 16C DNA nuclei were scarcely observed in the suspensor (Fig. 3h). In its both structural parts, nuclei during the fourth round of endoreplication dominated (approx. 35%), and slightly more than 20% of the nuclei contained 64C DNA. However, if we take into account the nuclei in or after the fifth round, those from the spherical part are by 2/3 more numerous than those from the elongated one. Again, however, in the spherical part of the suspensor, nearly 2/3 more of nuclei were at the higher level of ploidy (i.e., those nuclei that continued or had completed the fifth round of DNA endoreplication). Moreover, the first nuclei during the sixth round of DNA endoreplication appeared.

At the mid-cotyledon stage of the embryo proper, in the spherical part of the suspensor, the nuclei with higher degree of ploidy still dominated (Fig. 3i); nearly 40% of them were in the fifth endocycle, whereas, in the elongated part, nearly 50% of the nuclei were in the fourth endocycle.

At the advanced cotyledon stage, the nuclei containing 32 C DNA or less were not observed in the suspensor (Fig. 3j). In both structural parts, nuclei during and after the fifth round of replication dominated (by approx. 40 and 15%, respectively). At the same time, in the spherical part, still a significant proportion of nuclei was at the higher ploidy level, i.e., beginning or terminating the sixth round of endoreplication (20 and 6%, respectively).

### Simultaneous DNA endoreplication and amitotic nuclear divisions

The endoreplication process in the suspensor cells was correlated with amitotic nuclear divisions (Fig. 4a–f). In these nuclei, DNA synthesis started before the division was completed (Fig. 4d–f). It was evidenced by subtle EdU labeling of loose chromatin areas. Concurrently, no labeling was observed in large areas of condensed chromatin, representing probably late-replicating heterochromatin.

### Transcriptional activity in the suspensors at the selected developmental stages

**Fig. 5:**
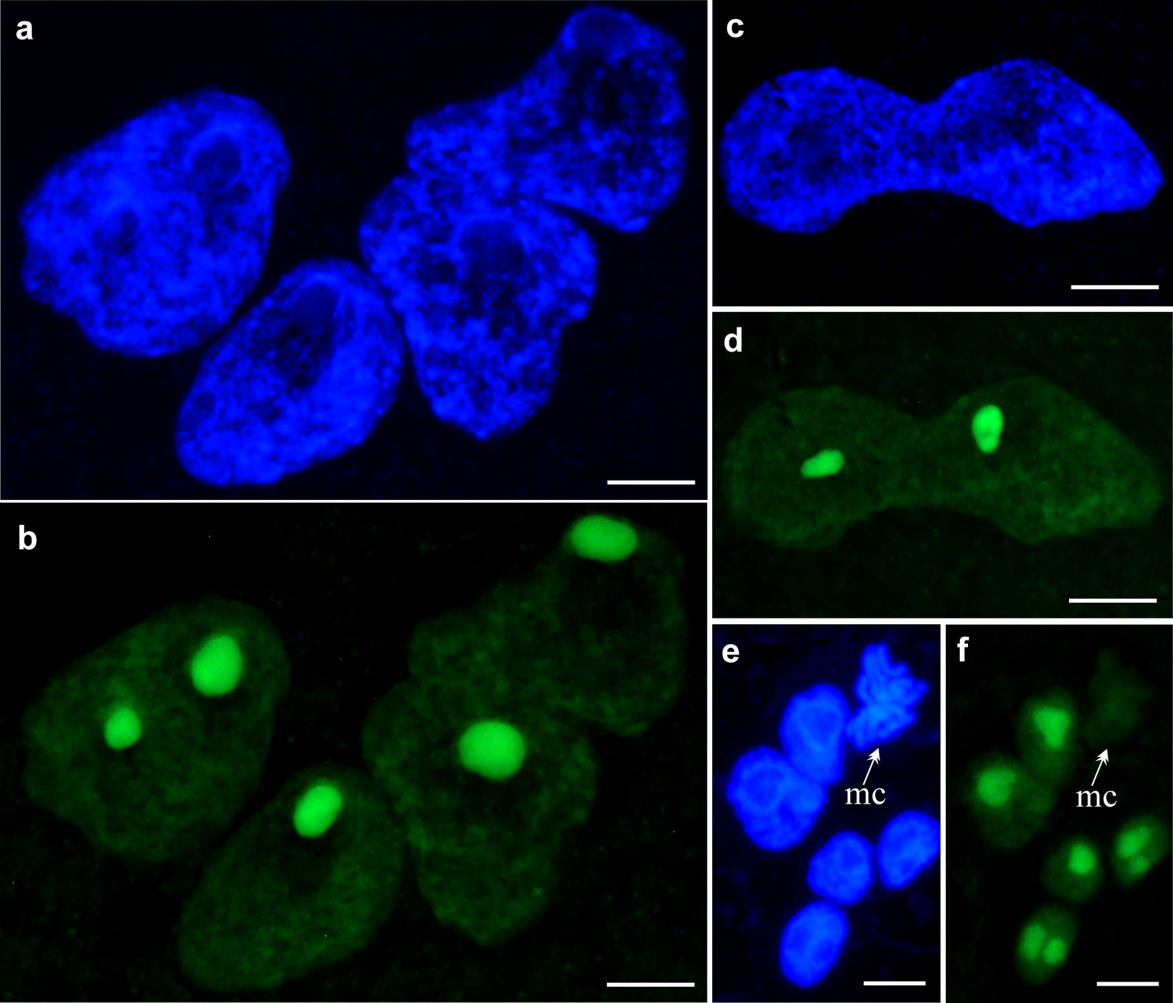
Detection of transcription in the nuclei of *P. sativum* var. Paloma suspensor. **a, c, e** DNA stained with DAPI in the polyploid nuclei (**a**), in the polyploid nucleus during the amitotic division (**c**), in the nuclei during classical cell cycle (**e**); mc—metaphase chromosomes. **b, d, f** The same nuclei as previously after 5-ethynyl uridine – (EU) incorporation stained with Click-iT^®^ RNA Alexa Fluor^®^ 488. Scale bar **a**–**d** − 50 µm; **e, f** − 10 µm

**Fig. 6:**
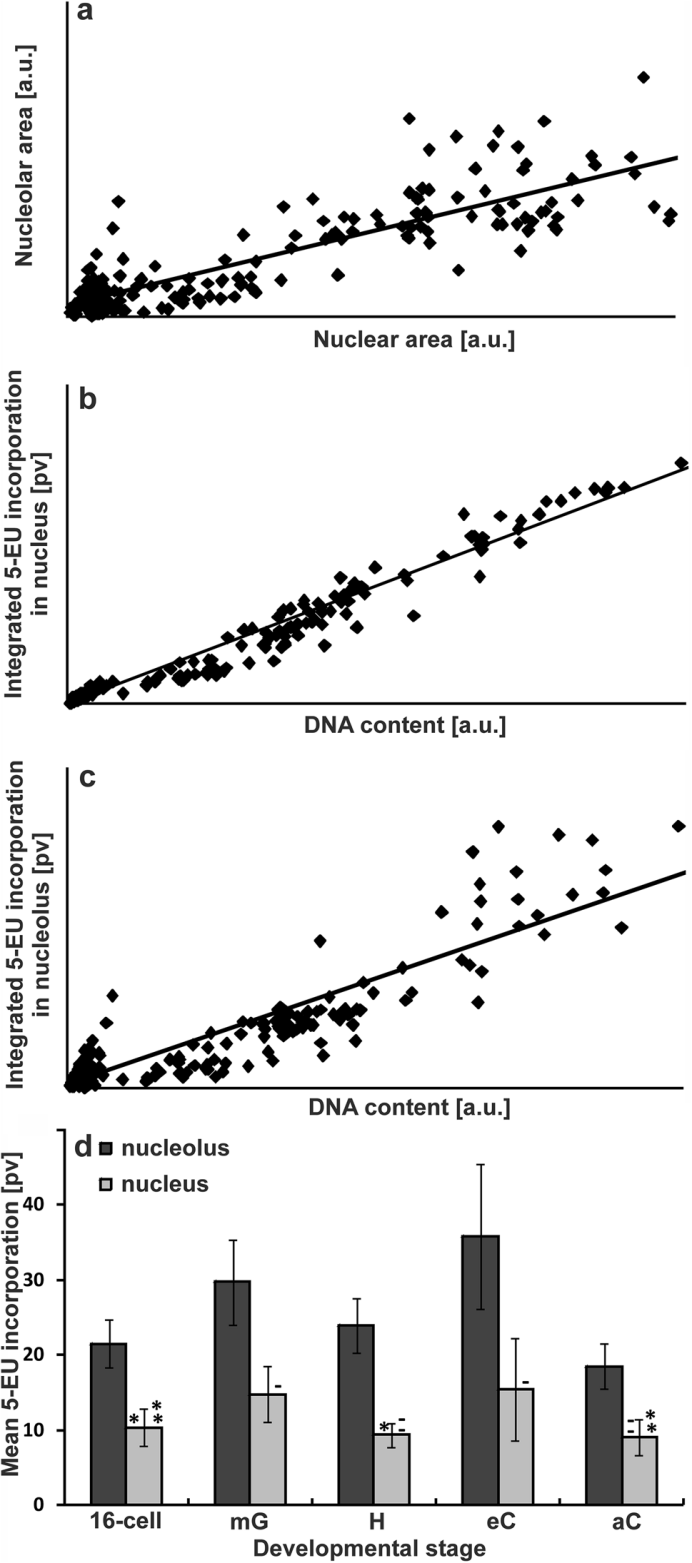
Amount of transcripts in the polyploidal nuclei of *P. sativum* var. Paloma suspensor. **a** Correlation between the area of polyploidal nuclei and area of their nucleoli *r* = 0.8931, *p* < 0.01; **b** Correlation between DNA content and integrated amount of transcripts in the nucleus *r* = 0.9839, *p* < 0.01; **c** Correlation between DNA content and integrated amount of transcripts in the nucleolus *r* = 0.9215, *p* < 0.01; amount of transcripts expressed as integrated fluorescence intensity; **d** Mean transcriptional activity in the nucleolar and perinucleolar chromatin regions of suspensors accompanying the embryo proper in the following stages of development: 16-cell, mid-globular—mG, heart—H, early cotyledon—eC, and advanced cotyledon—aC (**d**). Quantitative measurements of fluorescence were made after converting color images into grayscale and expressed in arbitrary units as mean pixel values (pv). The significance of differences in transcriptional activity were confirmed by Tukey’s test at *p* ≤ 0.05 (the pairs of results marked *), *p* ≤ 0.01 (the pairs of results marked **), and *p* ≤ 0.001 (others). The pairs of results where differences were statistically insignificant were indicated by (-) and (--). In each experimental series, five suspensors were analyzed and the studies were repeated three times

The analysis of the amount of 5-EU incorporated into newly synthetized RNA strands allowed to assess the transcriptional activity in the nuclei and nucleoli of the suspensor (Fig. 5). The suspensors attached to the embryo propers at the 16-cell, mid-globular, heart, early cotyledon, and advanced cotyledon stages were selected (Fig. 6), since they showed marked increases in DNA content, in contrast to the previous stages. In all the studied cases, a relatively higher level of fluorescence was observed at the nucleolar areas, compared with the perinucleolar regions of chromatin (Figs. 5, 6). During the amitotic divisions of suspensor nuclei, transcriptional activity was not switched off (as it happens during classic mitosis), but remained as high as it was in the nuclei which were not dividing (compare Fig. 5c–f).

The significant correlation between DNA content and the amount of transcripts is presented in Fig. 6a–c. It clearly indicates that with the increase in DNA content, thus also in the nucleus area, the nucleolar surface, and the amount of transcripts (expressed as integrated fluorescence intensity) both in the nucleolus and in the nucleus got bigger.

An increase in the level of polyploidization, however, did not enhance the average intensity of transcription in the nuclei (Fig. 6d). Despite the gradual increase in DNA content in the suspensor cells at the selected stages of embryo development (Fig. 3b, d, f, h, j), no corresponding gradual increase in average intensity of green fluorescence in the area of each particular nucleus was noticed (which could point to intensification of transcription activity in the certain areas of chromatin during suspensor development), but rather sinusoidal changes were found (Fig. 6d).

Initially, in the mid-globular stage, some increase in the transcriptional activity was observed in comparison with the previous 16-cell stage, and then, it dropped in the heart stage and was followed by next rise in the early cotyledon stage. The cotyledon stage was the longest one in the development of the *Pisum* embryo proper and was characterized by the highest transcriptional activity in the suspensor nucleoli. However, at this stage, despite the significant increase in DNA content (in comparison with the mid-globular stage; Fig. 3d, h), transcriptional activity (measured as mean fluorescence intensity) was only slightly more enhanced in the nucleoli and unchanged in the nuclei (Fig. 6d). At the final developmental stage of the suspensor (i.e., in an advanced cotyledon stage of the embryo proper, just prior to its degradation), transcriptional activity diminished significantly in the nucleolus to the lowest recorded value, while, in the nucleus, it was similar to that observed in the heart stage (Fig. 6d).

## Discussion

The suspensor development is often accompanied by nuclear endopolyploidization; however, endopolyploid nuclei are differently arranged inside them. A very high level of polyploidy was observed in the suspensors of *Tropaeolum majus* (2048C), *Phaseolus coccineus* (8192n), *Phaseolus vulgaris*, and *Phaseolus hysterinus* (4096n) (Brady 1973; Nagl 1976a, b; Kozieradzka-Kiszkurno et al. 2011; Henry and Goldberg 2015). In these plants with more complex suspensor structure, a gradient of nuclear polyploidy was also observed; it correlated with a gradient of cell size and cell differentiation on the chalazal–micropylar axis. A vast number of studies confirm the relationship between high ploidy of nuclei and the volume of the suspensor equipped with transfer walls and involved in the absorption of nutrients from the endosperm. A similar correlation was also observed in endosperm, in which highly polyploid nuclei are usually at the chalazal pole, in the place where many wall ingrowths are engaged in the absorption of nutrients from seed integuments (Brady 1973; Bohdanowicz 1997; Łuszczek et al. 2000; Kozieradzka-Kiszkurno et al. 2002, 2007; Kozieradzka-Kiszkurno and Bohdanowicz 2003; Świerczyńska and Bohdanowicz 2014; Henry and Goldberg 2015). In the four-celled suspensor of *P. sativum*, wall ingrowths are present on its whole surface, which maintains contact with the endosperm (Marinos 1970a, b). Nuclear DNA measurements in the suspensors of *P. sativum* var. Paloma showed that DNA endoreplication generally takes place with a certain similarity in their both structural parts. It begins very early, gradually increases during the development, and, in its final stage, creates nuclei with high ploidy level (128-256C), distributed both in the spherical and in the elongated parts of the suspensor. This phenomenon is consistent with the theory of a positive nuclear–cytoplasmic correlation. Accordingly, the increase in DNA content causes the increase in cell size, cytoplasm mass (number of organelles, membranes of the endoplasmic reticulum, ribosomes, etc.) and thereby enhances the absorption capacity of cells. Active transport also requires a significant energy input, which entails a significant increase in the number of mitochondria (D’Amato 1989; Biskup and Izmaiłow 2004; Lee et al. 2009; Perrot-Rechenmann 2010; Kozieradzka-Kiszkurno et al. 2011). Thus, the different arrangement of polyploid nuclei in the suspensors has its justification. However, the occurrence of polyploidy gradient in the micropyle–chalaza axis of expanded suspensors may indicate an additional role of endoreplication (Henry and Goldberg 2015). It seems possible that the gradient of DNA content contributes to forming a gradient of multiplied gene products for regulatory or signaling factors that may influence the dynamics and direction of flow of hormones or nutrient molecules, and thereby may influence the structural and metabolic changes of the embryo proper (Möller and Weijers 2009; Perrot-Rechenmann 2010; Wolpert et al. 2015). In *P. sativum* var. Paloma, in which the suspensor is quite large but composed of only two spherical and two elongated polynuclear cells (arranged in two layers), a characteristic regularity can also be noted. At the 8-cell stage of the embryo proper, endoreplication is first observed in the spherical part of the suspensor, adjacent to the embryo proper, and only later (in the 16-cell stage of the embryo proper), this process spreads to the elongated part of the suspensor. This phenomenon generates the periodic gradient of DNA content in the two layers of the suspensor. In the subsequent phases of the globular stage of the embryo proper, both structural parts of the suspensor reveal considerably similar DNA contents. When the embryo proper begins to change its shape from spherical- to heart-shaped, a further increase in DNA content is observed, first in the nuclei of the spherical part of the suspensor. A similar regularity also appears in the early and middle cotyledon stage, i.e., during intensive structural changes in the embryo proper. Thus, despite simple construction of the suspensor, its development creates a certain gradient of polyploidy, which occurs in more extensive suspensors. Consistency in the direction of DNA content changes implies that the signal about them is generated from the side of developing embryo proper and that, by feedback reaction, the temporarily appearing gradient of DNA content may indirectly influence the spatial structure of the embryo proper, according to the theory of a positive nuclear–cytoplasmic correlation, e.g., through, changes in the number of hormone transporters and amount of hormones at the way to and from the embryo proper (Larsson et al. 2008; Möller and Weijers 2009; Robert et al. 2013; Locascio et al. 2014; Liu et al. 2015; Wolpert et al. 2015; Peng and Sun 2018). However, the unequivocal confirmation of such correlation requires an additional research, and it will be extremely difficult, if only because of species-specific nature of the changes in DNA content. In *Vicia faba* having similarly built suspensor, at the globular stage of the embryo proper, the nuclei of suspensor do not reach 32C DNA, as is the case in *P. sativum*, but 8C DNA only. However, in the late heart stage of the embryo proper, the suspensor of *V. faba* with nuclei containing about 32-64C DNA undergoes degeneration (Borisjuk et al. 1995). In *P. sativum*, however, the suspensor continues to grow, reaching the highest degree of polyploidy (up to 256C in the spherical part and 128-256C in the elongated part) at an advanced cotyledon stage, which is followed immediately by its death.

The suspensor, like endosperm, develops prior to the embryo proper (Umehara and Kamada 2005). In *P. sativum* variety Paloma, four cells of the suspensor are formed when the embryo proper is composed of one or two cells. It is evident from our study that the acceleration of the suspensor growth and development is significantly effected by three phenomena showing some coincidence in large polyploid nuclei: (1) endoreplication, (2) transcription, and (3) amitotic divisions (Figs. 4d–f, 5c–f). We showed for the first time in plants that, in polyploid nuclei of the suspensor, DNA synthesis started before the amitotic division was completed. A similar phenomenon was probably observed only during amitotic division of polyploid haemopoietic stem cells present in the liver of urodeles (Barni et al. 1995). We also showed that, during division, transcriptional activity was not switched off, but remained as high as it was in the nuclei which were not dividing. Such modified course of events saves time in contrast to the normal cell cycle progression, during which DNA replication and nuclear division are separated by the G1 and G2 phases, while transcription is blocked by the mitotic chromosome condensation (Blagosklonny 2007). Thus, the rapid growth of the suspensor takes place not only by increase in DNA content and mass of cytoplasm, but also through rearrangement of the order of the key processes.

An increase in DNA content (via endocycles) may significantly enhance transcription and improve cell metabolism due to gene multiplication (Świerczyńska and Bohdanowicz 2014; Henry and Goldberg 2015). As it was demonstrated in the current study, the increase in DNA content in *P. sativum* var. Paloma was correlated with the greater amount of transcripts. However, this correlation does not explain why the transcriptional activity is changed in the suspensor, and whether the increased DNA content results in the upregulated transcriptional activity. The possible link between them is promising but requires further evidence. It is believed that an increased transcription and metabolic activity in the suspensor are positively correlated with the production of plant hormones (Umehara and Kamada 2005; Henry and Goldberg 2015). For example in the giant suspensors of *Phaseolus coccineus, Phaseolus vulgaris, Tropaeolum majus*, and *Cytisus laburnum*, the levels of mRNA encoding enzymes of hormone biosynthetic pathway as well as hormones (GA and IAA), are higher than in the cells of the embryo proper (Solfanelli et al. 2005; Kawashima and Goldberg 2010; Henry and Goldberg 2015). However, high transcriptional activity connected with hormone production is species-specific, and the biosynthesis of hormones (e.g., GA) is not a common feature of all plant suspensors. In *P. sativum*, hormones are synthesized in endosperm and embryo proper (Brenner 1987; Swain et al. 1995). In addition, in *Arabidopsis thaliana*, the *GA3-ox1-3* genes required for the synthesis of gibberellic acid are inactive in the suspensor and mRNA coding GA3-oxidase does not accumulate therein, but in the endosperm (Belmonte et al. 2013). Thus, it is possible that the massive suspensors, such as in *Phaseolus*, take up part of the functions performed by an endosperm (which, in these species, is much smaller and has delayed cellularization), and therefore, they have the higher metabolic activity associated with the production of hormones, whereas, in the less extensive suspensor of *Pisum*, endoreplication is needed mainly for the intensification of absorption and transport of metabolites from endosperm to the developing embryo proper.

The multiplication of DNA copies in *P. sativum* suspensor, however, does not lead to linear increase in the average intensity of transcription in the area of particular nuclei at the subsequent stages of development. This is probably because polyploidy can substantially alter the transcriptome not only by changing transcription intensity proportional to overall genome content, but also polyploidization is commonly accompanied by uneven alterations of locus-specific transcription, driven partly by epigenetic changes. However, polyploidy itself was also found to impact plasticity of those epigenetic changes (Schoenfelder and Fox 2015). Furthermore, each endoreplication creates unique genomic deletions and rearrangements, generating sequence heterogeneity, both at the same locus within nucleus, and among different nuclei. Underreplicated or overreplicated regions can exhibit decreased or increased gene expression, respectively (Yarosh and Spradling 2014; Schoenfelder and Fox 2015).

The increase in DNA content appearing at an early stage of suspensor development and, consequently, the resulting changes at the level of transcription are probably the main factors determining the maintenance of the suspensor identity.

### Author contribution statement

JP and ACh conceived and designed research. ACh, AŻ, and JP conducted experiments. ACh, JP, and KK analyzed data. ACh and KK conducted statistical analysis. JP wrote the manuscript. JM conducted a language correction. All authors read and approved the manuscript.
